# Targeting PARP proteins in acute leukemia: DNA damage response inhibition and therapeutic strategies

**DOI:** 10.1186/s13045-022-01228-0

**Published:** 2022-01-22

**Authors:** Antonella Padella, Andrea Ghelli Luserna Di Rorà, Giovanni Marconi, Martina Ghetti, Giovanni Martinelli, Giorgia Simonetti

**Affiliations:** 1 Biosciences Laboratory, IRCCS Istituto Romagnolo Per Lo Studio Dei Tumori (IRST) “Dino Amadori”, Meldola, FC Italy; 2 Hematology Unit, IRCCS Istituto Romagnolo Per Lo Studio Dei Tumori (IRST) “Dino Amadori”, Meldola, FC Italy; 3 Scientific Directorate, IRCCS Istituto Romagnolo Per Lo Studio Dei Tumori (IRST) “Dino Amadori”, Meldola, FC Italy

**Keywords:** PARP, Acute myeloid leukemia, Acute lymphoblastic leukemia, Synthetic lethality, Biomarkers, DNA damage response, Preclinical studies, Clinical trials

## Abstract

The members of the Poly(ADP‐ribose) polymerase (PARP) superfamily are involved in several biological processes and, in particular, in the DNA damage response (DDR). The most studied members, PARP1, PARP2 and PARP3, act as sensors of DNA damages, in order to activate different intracellular repair pathways, including single-strand repair, homologous recombination, conventional and alternative non-homologous end joining. This review recapitulates the functional role of PARPs in the DDR pathways, also in relationship with the cell cycle phases, which drives our knowledge of the mechanisms of action of PARP inhibitors (PARPi), encompassing inhibition of single-strand breaks and base excision repair, PARP trapping and sensitization to antileukemia immune responses. Several studies have demonstrated a preclinical activity of the current available PARPi, olaparib, rucaparib, niraparib, veliparib and talazoparib, as single agent and/or in combination with cytotoxic, hypomethylating or targeted drugs in acute leukemia, thus encouraging the development of clinical trials. We here summarize the most recent preclinical and clinical findings and discuss the synthetic lethal interactions of PARPi in acute myeloid leukemia (AML) and acute lymphoblastic leukemia (ALL). Despite the low frequency of genomic alterations of *PARP* and other DDR-related genes in acute leukemia, selective vulnerabilities have been reported in several disease subgroups, along with a “BRCAness phenotype.” AML carrying the *RUNX1-RUNX1T1* or *PML-RARA* fusion genes or mutations in signaling genes (*FLT3*-ITD in combination with *TET2* or *TET2* and *DNMT3A* deficiency), cohesin complex members (*STAG2*), *TP53* and *BCOR* as co-occurring lesions, *IDH1/2* and ALL cases expressing the *TCF3-HLF* chimera or *TET1* was highly sensitive to PARPi in preclinical studies. These data, along with the warning coming from the observation of cases of therapy-related myeloid malignancies among patients receiving PARPi for solid tumors treatment, indicate that PARPi represents a promising strategy in a personalized medicine setting. The characterization of the clonal and subclonal genetic background and of the DDR functionality is crucial to select acute leukemia patients that will likely benefit of PARPi-based therapeutic regimens.

## Background

The Poly(ADP‐ribose) polymerase (PARP) superfamily is composed by 17 proteins involved in crucial biological processes such as DNA damage response (DDR), transcription, chromatin structure stabilization, cell metabolism, telomere length maintenance, antiviral response and cell signaling (Table 1) [1]. So far, eight PARP proteins (PARP1, PARP2, PARP3, PARP5, PARP5b, PARP6, PARP9 and PARP14) have been recognized as key factors involved in the maintenance of genetic stability as they control DNA damage repair and cell cycle regulation [2]. PARP proteins act as mediator of the initial phases of the response to DNA damages. Indeed, they interact with DNA damaged sites [3, 4] and promote the recruitment of additional DNA repair proteins [5]. To facilitate the localization of DDR proteins to the site of damage, PARP proteins transfer ADP-ribose residues from NAD^+^ to target substrates (DNA or proteins) [6]. The majority of PARPs enzymes (*n* = 12) create mono-(ADP-ribose) modification on their targets (Mono-ADP-Ribose [MAR]ylation), and four of them extend the initial modification site to form poly(ADP-ribose), or PAR chains (PARylation) [7]. This post-translational modification regulates the conformation, stability and activity of the targeted proteins. The generation of PAR chains is crucial to promote DNA damage repair thorough two different mechanisms: (1) the rapid and efficient PAR-dependent recruitment of DNA repair factors and histones on the site of the damage [5], (2) the PARylation of DNA repair factors that consequently promotes their association to the DNA and their interaction with other proteins involved in the repair cascade [8].

**Table 1:** Biological function and enzymatic activity of PARP proteins in eukaryotic cells

PARP	Enzymatic activity	Biological function	References
PARP1	Poly-	DDR	[1]
PARP2	Poly-	DDR	[1]
PARP3	Mono-	DDR and mitosis regulation	[9]
PARP4	Poly-	Antiviral response	[10]
TNSK1 PARP5a	Poly-	DDR, telomere maintenance and mitosis regulation	[11]
TNSK2 PARP5b	Poly-	DDR, telomere maintenance and mitosis regulation	[12]
PARP6	Mono-	Cell cycle progression	[13]
PARP7	Mono-	Cell–cell adhesion, inhibition of type I interferon response and gene regulation	[14]
PARP8	Mono-	Unknown	–
PARP9	Inactive	DDR, gene transcription and antiviral response	[15]
PARP10	Mono-	binding protein and an inhibitor of MYC with inhibitory potential also on the NF-κB signaling pathway	[16, 17]
PARP11	Mono-	Role in nuclear envelope biology	[17]
PARP12	Mono-	Regulation of stress granule assembly, microRNA activity and antiviral response	[18, 19]
PARP13	Inactive	Regulation of microRNA activity	[20]
PARP14	Mono-	Survival, cell migration, assembly of stress granules, transcription during inflammation processes, DDR and antiviral response	[21, 22]
PARP15	Mono-	Regulation of stress granule and antiviral response	[10, 22]
PARP16	Mono-	Regulation of unfolded protein response	[23, 24]

DDR: DNA damage response

This review aims to discuss the potentials of PARP inhibitors (PARPi)-based therapeutic strategies in acute leukemia. To this aim, we first summarize the role of PARP proteins in the DDR and the mechanisms of action of PARPi, which helps understanding their preclinical and clinical successes and failures in acute leukemias. We then focus on synthetic lethal interactions of PARPi in acute myeloid leukemia (AML) and acute lymphoblastic leukemia (ALL), which opens a promising therapeutic window for specific disease subgroups.

## PARP enzymes in the response to DNA damages

PARP1, PARP2 and PARP3 are the most studied enzymes for their involvement in the DDR. In mammalian cells, they act as DDR sensors in response to different types of DNA damages. In particular, PARP1 responds to single-strand breaks (SSBs), DNA cross-links, stalled replication forks and double-strand breaks (DSBs) [25]. PARP2 seems to recognize more specifically gaps and flap structures [26], while PARP3 was described to respond more selectively to DSBs [9, 27]. Briefly, PARP1, PARP2 and PARP3 cooperate with several mediators in the DDR through the activation of different intracellular repair pathways such as single-strand repair (SSR), homologous recombination (HR), conventional non-homologous end joining (cNHEJ) and alternative non-homologous end join (aNHEJ). The following sections summarize the role of PARP proteins and, in particular of PARP1, in the response to SSBs and DSBs and their role in the activation of different repair pathways.

### PARPs in base excision repair and single-strand breaks repair

**Fig. 1:**
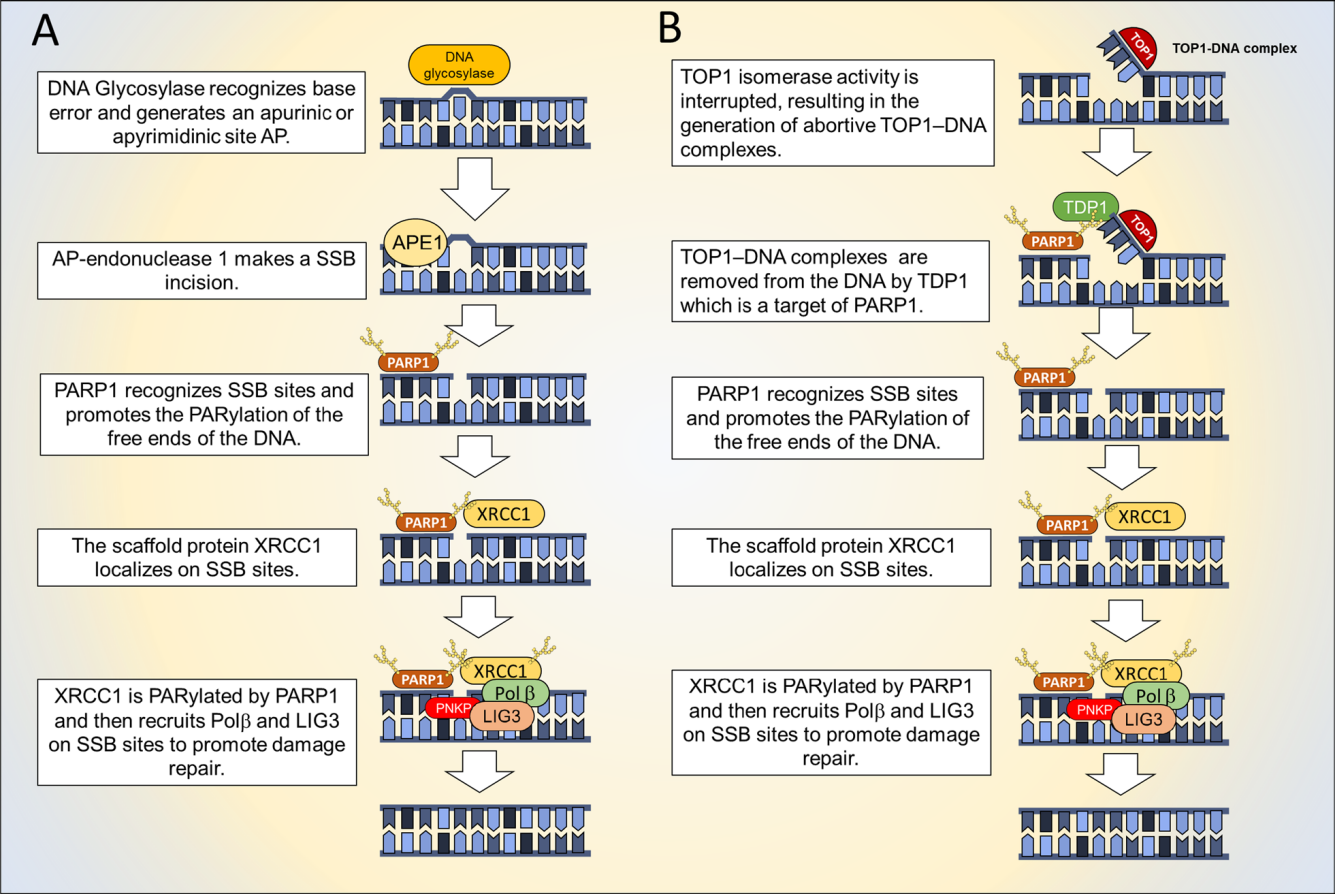
Mechanism of action of PARP1 in base excision repair (**A**) and single-strand DNA nick repair (**B**)

Base modifications can arise by endogenous sources or errors during DNA replication and both PARP1 and PARP2 enzymes play a central role in their repair throughout base excision repair/single-strand break repair process (BER/SSBR) [28]. PARP1 PARylates a variety of substrates, thus promoting the accumulation repair factors, which in turn interact with single-strand DNA (ssDNA) and act as scaffold for other repair factors [29–31]. At first, the mismatched base is cleaved by a DNA glycosylase generating an apurinic/apyrimidinic site (AP, Fig. 1A). Then, apurinic/apyrimidinic Endonuclease 1 (APE1) removes the AP site generating a ssDNA nick which is recognized and processed as a SSB by PARP1[32]. In details, after PARylation, PARP1 interacts with proteins such as DNA polymerase β (Polβ), DNA ligase III (LIG3), X‐ray repair cross‐complementing protein 1 (XRCC1) and bifunctional polynucleotide kinase 3′-phosphatase (PNKP) which are recruited at the SSB site in the repair process[33]. Another type of single base DNA lesion, ssDNA nicks, require the activity of PARP1 to be repaired. ssDNA nicks are generated by the deregulated activity of DNA topoisomerase 1 (TOP1) [34]. Indeed, TOP1 relaxes topological stress in the DNA structure by generating a cut on one DNA strand, controlling its rotation around the intact strand and, finally, removing the nick (Fig. 1B). The entire process can be interrupted, resulting in the generation of abortive TOP1–DNA complexes (TOP1 cleavage complexes, TOP1cc). TOP1cc is removed from the DNA by tyrosyl-DNA phosphodiesterase 1 (TDP1) which is a target of PARP1 [35, 36]. The exposed nick can be a substrate for SSBR [36].

### PARPs in double-strand breaks repair

**Fig. 2:**
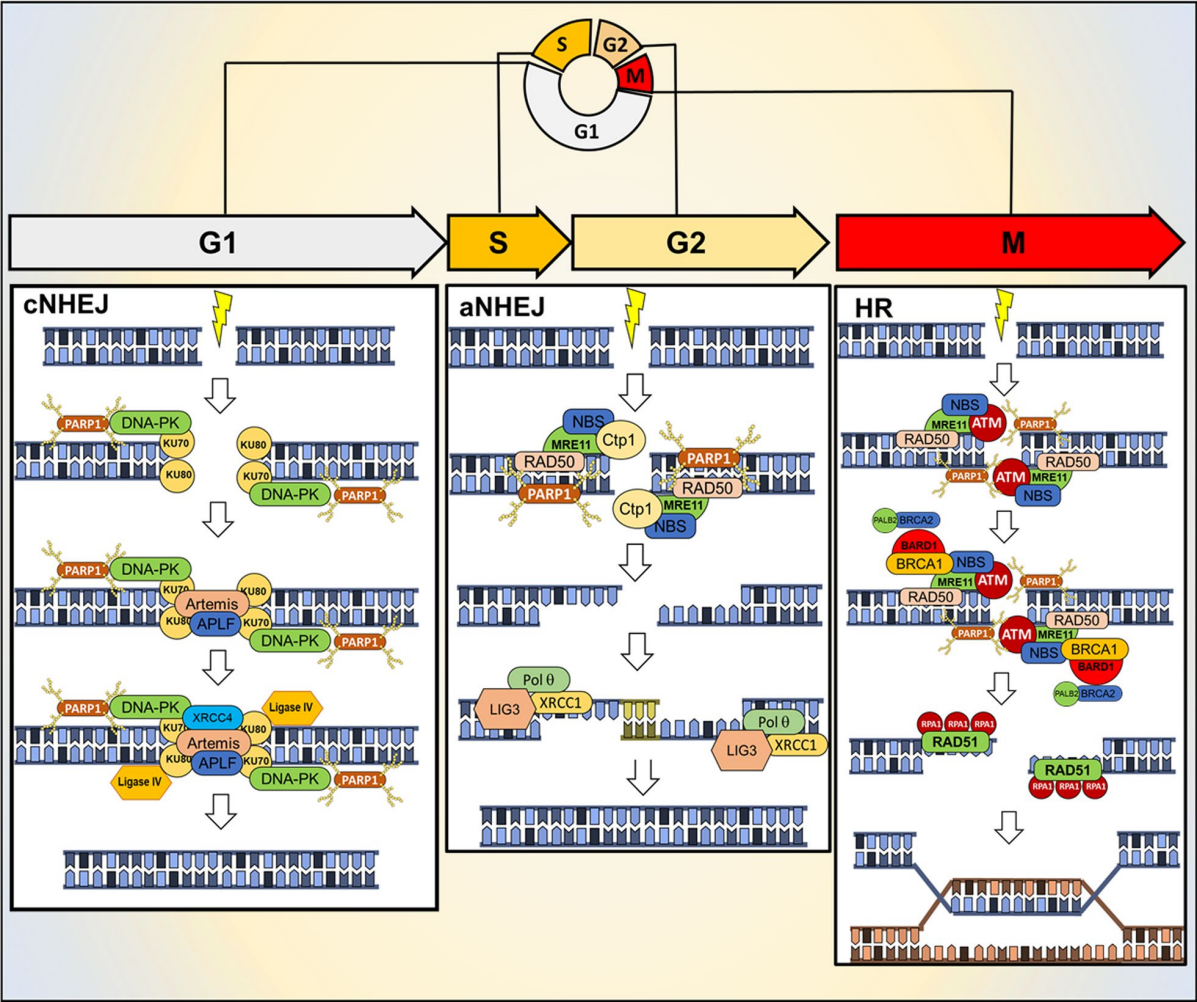
Mechanism of action of PARP1 in cNHEJ, aNHEJ and HR repair according to the cell cycle phases

In eukaryotic cells, DSBs are repaired through HR, cNHEJ or aNHEJ repair pathways, according to the cell cycle phase [37]. PARP1 plays a critical role in DSB sensing and its recruitment and activation occur within 100 ms after introduction of DSBs [38]. Thus, PARP-1 activation is one of the earliest events in the sensing of DSBs. Independently of the downstream pathway, PARP1 recognizes DSBs and responds to it by recruiting the initial mediators of the DSBs repair response. The choice between the different DNA repair pathways is related to the cell cycle phases (as already mentioned), type of DNA damages, organism and to the proficiency of these pathways [39, 40]. It has been showed that cNHEJ and aNHEJ repair systems repair more efficiently DSBs in comparison with HR [39]. In eukaryotic cells with complex genome, the HR system is preferentially use to repair DSBs that can be generated during DNA replication [41]. The following sections briefly summarize the role of PARP1 in these three repair pathways (Fig. 2).

#### PARP1 in conventional non-homologous end joining repair

cNHEJ is the chosen mechanism of DDR during interphase (G0/G1 in particular) [42]. PARP1 PARylates the DNA-dependent protein kinase catalytic subunit (DNA-PKcs), which is a crucial NHEJ factor [43] (Fig. 2A). PARylation stimulates the kinase activity of DNA-PKcs and the requirement of the KU70–KU80 complex [44]. KU70-KU80 complex promotes the localization and activation of the DNA ligase IV/XRCC4 complex on DNA end. Before DNA ligase IV ligation, DNA ends need to be processed by the concomitant action of the nuclease Artemis and aprataxin-polynucleotide kinase-like factor (APLF). After this process, DNA ligase IV complex with XRCC4 and XRCC4-like factor (XLF) to rejoin the DNA ends [45].

#### PARP1 in alternative non-homologous end joining repair

PARP1 has a crucial role in the regulation of aNHEJ repair, that is mostly active in S and G2 phases of cell cycle [46, 47] (Fig. 2B). In addition, the cell may also use this pathway during G1 phase to respond to DSBs [48, 49]. PARP1 is involved in the first steps of the repair process and, in particular, it promotes the localization of the MRN-CtIP complex on the DNA ends [50]. The CtIP promotes the endo/exonuclease activity of MRN resulting in exposition of microhomology sequences of ssDNA [51]. Then, PARP1, MRN complex and the DNA polymerase θ (Polθ) promote the bridging and alignment of the DNA single strands via the microhomology sequences. The 3' sequences that have no homology are digested by ERCC1 and XPF nucleases. Finally, the generated gaps are filled by Polθ-mediated DNA synthesis and the remaining nicks are repaired by the LIG3-XRCC1 complex [52, 53].

#### PARP1 in homologous recombination repair

After DNA replication, DSs are preferentially repaired through the HR repair [54]. PARP1 promotes the recruitment and activation of different downstream partners during the process (Fig. 2C), including the MRE11-RAD50- NBS1 (MRN) complexes, which is involved in the response to DSBs via HR [55]. PARP1 is important for the early and rapid recruitment of BRCA1 to DSBs. BRCA1 function is to promote DSB ends resection [56–58]. Different studies have highlighted that BRCA1 can be loaded to DSBs independently of PARP1 activity and, mainly, following DNA damage-mediated ubiquitylation [59]. The localization of BRCA1 to DSBs is enhanced by the PARylation of BRCA1-associated RING domain protein 1 (BARD1) on the site of damage. BRCA1-BARD1 complex promotes the endonuclease activity of MRE11 which generates single-strand DNA on the site of damages [60, 61]. Resected DNA is a substrate for RAD51 binding, but it is initially bound by the replication protein A (RPA), requiring mediator proteins to assist RAD51 loading onto single-strand DNA. RAD51 promotes strand-exchange of single-strand DNA filament that invades an unbroken homologous DNA which is typically the sister chromatid [62]. The generation of single-strand DNA is fundamental for the final steps of HR repair and in particular for the mechanism of strand invasion which promotes DSBs repair [63]. The resection activity of the complex BRCA1-BARD1-MRE11 is limited by the activity of PARP1 which promotes the PARylation of BRCA1 and the association of the receptor-associated protein 80 (RAP80). The interaction with RAP80 stabilizes BRCA1 and suppresses HR, which results in limited strand invasion and in the subsequent repair of the DSB.

## Genomic alterations of *PARPs* and DDR genes interactors in acute leukemia

### *PARP* genomic alterations in acute leukemia

**Fig. 3:**
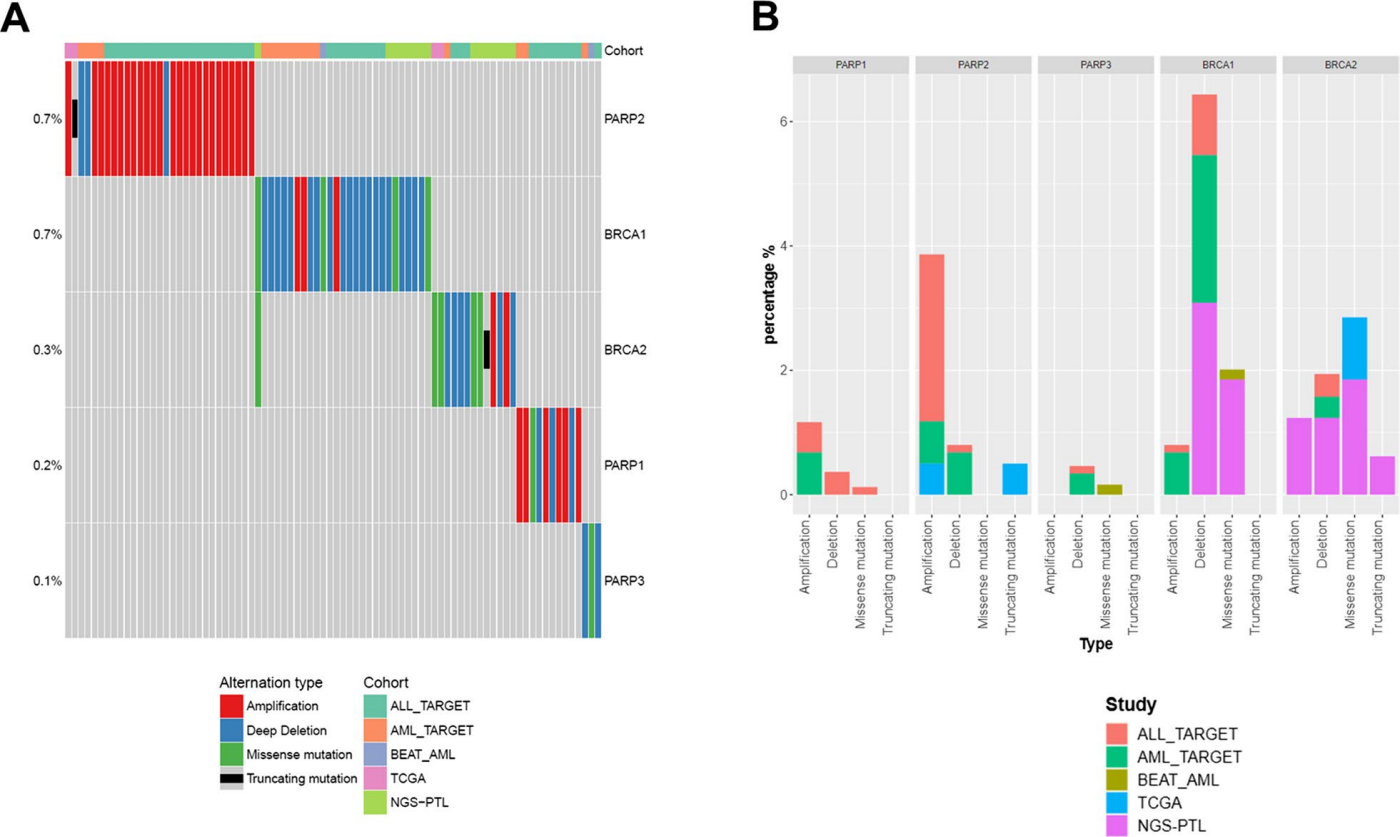
Genomic alterations of *PARP1/2/3* and *BRCA1/2* in pediatric AML and ALL TARGET study and adult AML cohorts (Beat AML, TCGA-LAML and NGS-PTL). **A** Oncoprint of genomic alterations of *PARP1, PARP2, PARP3, BRCA1* and *BRCA2* acute leukemia cohorts. **B** Frequency and type of genomic alterations

According to public genomic data available on acute leukemias on the cBio portal [64] (TCGA-LAML [65] and Beat AML [66] datasets) and our cohort (NGS-PTL [67, 68]), there is no evidence of genomic alterations of *PARP1* in adult patients and few cases have been reported in pediatric cohorts [64] (2/295 in TARGET-AML [69], 0.7%; 8/819 in TARGET-ALL [70], 1.0%, Fig. 3A and Table 2). Conversely, alterations have been reported in *PARP2*: 2.7% of pediatric ALL cases showed gene amplifications (22/819 patients, 2.7%) or deletions (1/819 patients, 0.1%), while they accounted for 1.4% of pediatric AML cases (2/295 patients with deletions, 0.7% and 2/295 patients with amplification, 0.7%). In adults, *PARP2* was found mutated in one case and amplified in another one in the TCGA-LAML cohort (2/200 patients, 1.0%). *PARP3* was deleted in two cases of pediatric AML and ALL (1/819 ALL TARGET and 1/295 AML-TARGET, 0.1% and 0.3%, respectively) and mutated in one patient from the Beat AML cohort (1/622, 0.2%).

**Table 2:** List of alterations in *PARP1, PARP2, PARP3, BRCA1* and *BRCA2* genes in acute leukemia cohorts

Study ID	Number of patients	Type of alteration	Number of patients with the alteration	Percentage	Gene
*Pediatric cohorts*					
TARGET-AML	295	AMP	2	0.7	*PARP1*
TARGET-AML	295	AMP	2	0.7	*PARP2*
TARGET-AML	295	DEL	2	0.7	*PARP2*
TARGET-AML	295	DEL	1	0.3	*PARP3*
TARGET-AML	295	AMP	2	0.7	*BRCA1*
TARGET-AML	295	DEL	7	2.4	*BRCA1*
TARGET-AML	295	DEL	1	0.3	*BRCA2*
TARGET-ALL	819	AMP	4	0.5	*PARP1*
TARGET-ALL	819	DEL	3	0.4	*PARP1*
TARGET-ALL	819	Missense mutation [E883Q]	1	0.1	*PARP1*
TARGET-ALL	819	AMP	22	2.7	*PARP2*
TARGET-ALL	819	DEL	1	0.1	*PARP2*
TARGET-ALL	819	DEL	1	0.1	*PARP3*
TARGET-ALL	819	AMP	1	0.1	*BRCA1*
TARGET-ALL	819	DEL	8	1	*BRCA1*
TARGET-ALL	819	DEL	3	0.4	*BRCA2*
*Adult cohorts*					
Beat AML	622	Missense mutation [T403I]	1	0.2	*PARP3*
Beat AML	622	Missense mutation [K251E]	1	0.2	*BRCA1*
TCGA-LAML	200	AMP	1	0.5	*PARP2*
TCGA-LAML	200	Truncating mutation [R150*]	1	0.5	*PARP2*
TCGA-LAML	200	Missense mutation [I3312K; V295I]	2	1	*BRCA2*
NGS-PTL-AML	162	DEL	5	3.1	*BRCA1*
NGS-PTL-AML	162	Missense mutation [R496C; N1132D; A1669S]	3	1.9	*BRCA1*
NGS-PTL-AML	162	AMP	2	1.2	*BRCA2*
NGS-PTL-AML	162	DEL	2	1.2	*BRCA2*
NGS-PTL-AML	162	Missense mutation [G1771D; S384F]	3	1.9	*BRCA2*
NGS-PTL-AML	162	Truncating mutation [N213fs]	1	0.6	*BRCA2*

AMP: copy number amplification; DEL: copy number deletion

Overall, the genomic lesions preferentially include amplifications of the *PARP2* gene (Fig. 3B), with potential gain of function consequences providing a selective advantage to the malignant cells in terms of ability to maintain a tolerable dose of genetic instability.

### Genomic alterations of *BRCAs* and other DDR genes in acute leukemia

Mutations, copy number alterations and/or polymorphisms of other DDR genes have been described, that may result in the deregulation of the HR pathway activity. Mutations in the *PARP1/2/3* downstream partners *BRCA1/2* genes have been reported in adult AML, with a frequency around 1.9% in the NGS-PTL AML cohort (*BRCA1*: 3/162 cases, *BRCA2*: 4/162 cases) and below 1.0% in the Beat AML study (*BRCA1*: 1/622 cases; *BRCA2*: 2/200). Deletions, missense and truncating mutations, that likely induce loss of function phenotypes, are the most recurrent alterations in *BRCA1* and *BRCA2* genes (Table 2), as expected based on the role of the encoded proteins [71, 72]. The presence of polymorphisms in the HR genes *RAD51 (*135C*)* and its paralog *XRCC3* (241M) has been associated with an increased risk of de novo and t-AML [73]. Deletion of *Mre11A* and *ATM* on chromosome 11 has been described in t-AML patients and leads to alterations in both NHEJ and HR as Mre11A is an early factor in these two pathways [74]. Alterations in the Fanconi Anemia (FA) pathway have also been observed, which, as first consequence, results in the development of FA, but about 9% of patients subsequently develop AML with a high incidence of chromosomal breakage [75]. Heterozygous deletions and distinct point mutations in the *FANCA* gene were found in a small percentage of AML patients [76] and have been associated with those cases carrying chromothripsis [77]. In T-ALL, a *FANCC* point mutation was identified [78]. Genomic alterations in other DDR genes such as *ATM*, *PRKDC*, *ATR*, *RPA1*, *DSS1*, *NBN*, *RAD51*, *RAD54*, *CHEK1*, *CHEK2*, *ERCC1*, *POLB*, *FEN1* and *CDK12* have shown synthetic lethality in combination with PARPi [79] and are altered at a frequency ≤ 1% in acute leukemias [64].

Moreover, we have recently reported that about 5% of AML cases carry heterozygous copy number loss of *PALB2,* a gene of the FA pathway that interacts with BRCA1 and BRCA2 in the DNA damage repair [80]*.* Patients with *PALB2*-mutated solid tumors have been reported to be responsive to PARPi [81–83], and a phase II clinical trial showed that olaparib is an effective treatment for patients with metastatic breast cancers carrying germline mutations in *PALB2* [84]*.* In addition, another phase II trial is enrolling patients with advanced HER2-negative breast cancer and other solid tumors with mutations in HR genes (excluding *BRCA1/2*) for the evaluation of the treatment with talazoparib (NCT02401347). Therefore, *PALB2* deletion is a potential marker of HR defects*,* that may also suggest a potential vulnerability to PARP inhibition in AML.

## PARP inhibitors: mechanism of actions

### Inhibition of single-strand breaks and base excision repair

**Fig. 4:**
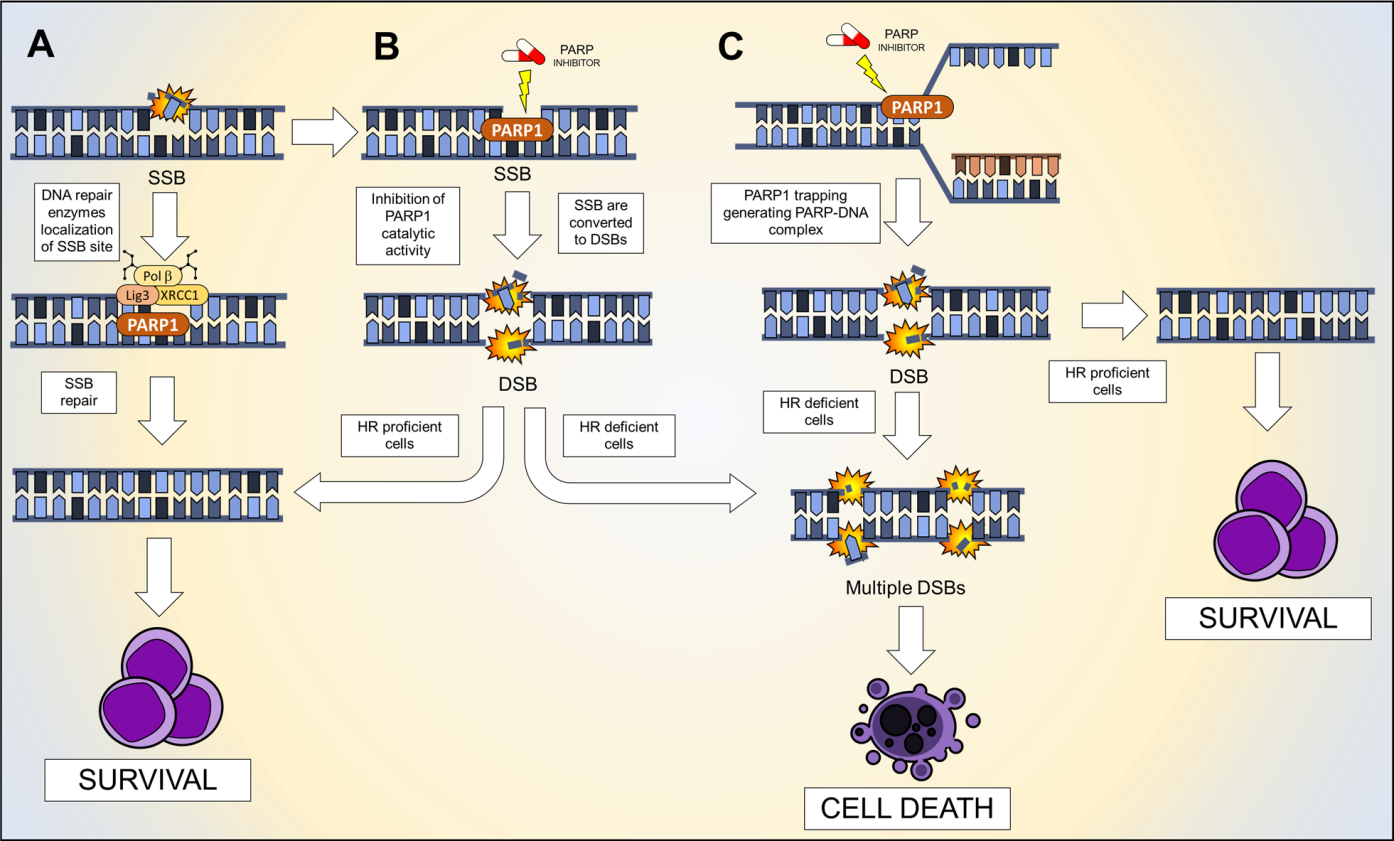
Schematic representation of PARPi mechanism of action. **A** SSBs are normally identified and repaired by PARP1 and **B** the addition of a PARPi compromises the repair and the SSB is converted in DSBs. **C** PARP1 inhibitors can generate PARP-DNA complexes that during DNA replication can promote DNA replication forks collapse and, consequently, the generation of DSBs. The fate of DSBs depends on HR proficiency of the cancer cells. If the cells are HR proficient, DSBs are repaired and the cells survive, on the contrary, DSBs are not repaired, damages accumulate during replication until cancer cells’ death

As already mentioned, SSBs arise frequently in proliferating cells and they are repaired through PARP-dependent DNA repair mechanisms (e.g., BER). Efficient SSB repair is essential for the survival of proliferating cells (Fig. 4A). All the developed PARPi act on the catalytic activity of PARP enzymes and, thus, on the repair of SSBs by BER. As a consequence, unrepaired SSBs can be converted to the more cytotoxic DSBs that, if not repaired, cause cell death [85] (Fig. 4B).

### PARP trapping and generation of PARP-DNA complexes

As an additional mechanism of action, PARPi can trap PARP1 and PARP2 enzymes on DNA [86] (Fig. 4C). It has been showed that PARP1 is the crucial factor in DNA trapping by PARPi, as its depletion, rather than PARP2 depletion, reduces the sensitivity to PARP inhibition [86]. PARP entrapment can occur on DNA strand break sites as well as TOP1-processed ribonucleotides or on Okazaki-fragment intermediates during DNA replication [87]. Once trapped, PARP1 cannot dissociate from DNA due to inhibition of its catalytic activity, which is required to release PARylated PARP1 from the DNA. PARP1-DNA complexes are highly toxic for replicating cells. Firstly, they block DNA replication by interfering with the protein complexes at replication forks [88]. This event can culminate in the collapse of replicative forks and the generation of DSBs. Moreover, these processes prevent the accessibility of other repair proteins to the damaged sites [89–91]. While catalytic inhibitory effects of the clinically available PARPi are similar, their ability in trapping PARP–DNA complexes varies considerably. Talazoparib exhibits the highest trapping efficiency while Veliparib the lowest (Table 3).

**Table 3:** Summary of the available PARP inhibitors with primary and secondary molecular targets and their efficacy in PARP trapping

Compound	Primary target(s)	Secondary target(s)	PARP trapping
Olaparib/AZD-2281/KU005436	PARP1	PARP2/3/4	***
Rucaparib/ Rubraca/AG-0146991	PARP1/2	PARP2/3/4/10, TNSK1/2	**
Veliparib/ABT-888	PARP1/2		*
Niraparib/MK-4827	PARP1/2	PARP3/4/12	****
Talazoparib/BMN-673	PARP1/2/3	PARP4, TNSK1/2	*****

*PARP trapping strength. Stars denotes the intensity trapping, form lower (*) to higher (*****) intensity

### Immunomodulatory activity of PARPi in acute leukemia

In addition to a direct activity on the DDR pathway, PARPi exert an immunomodulatory function, as demonstrated in multiple cancer types. Numerous lines of evidence now suggest that the DDR plays an important role in driving sensitivity and response to immune checkpoint blockade [92]. First, DDR dysfunction, and in particular HR deficiency, can increase the tumor mutational burden [93], which in turn can lead to the generation of neoantigens, and in particular mutation-associated neoantigens can enhance anticancer T cell activity [94]. Second, S phase-specific DNA damages in cells with DDR-related gene alterations or under treatment with PARPi result in the activation of the stimulator of interferon genes pathway, that promotes T cell infiltration and activity [95, 96], but is also associated with upregulation of immunosuppressive PD-L1 expression [97]. Therefore, PARPi, by enhancing tumor immunogenicity through increased tumor mutational burden, neoantigen release and PD-L1 expression, create the ideal microenvironment for combined treatments with immunostimulatory drugs [98]. This evidence, which comes from solid tumors, deserve further investigation in acute leukemias. Moreover, PARPi can counteract immune escape in AML. Malignant cells in monocytic leukemia, but not in myeloblastic and immature disease subtypes, produce reactive oxygen species (ROS) through NADPH oxidase and in turn induce PARP1-dependent apoptosis of natural killer (NK), CD4^+^ T and CD8^+^ T cells [99]. In parallel, genotoxic stress can induce the upregulation of NKG2D ligands on tumor cells, a process mediated by ATM [100]. Binding of NKG2D ligands to the NKG2D receptor on the surface of NK cells and activated CD8^+^ T cells functions as a costimulatory signal. Paczulla and colleagues showed that PARP1 represses NKG2D ligands expression in leukemia stem cells, thus contributing to their selective escape from immune surveillance by NK cells [101]. Accordingly, PARPi induced the expression of NKG2DLs on the surface of AML cells, but not on healthy hematopoietic stem and progenitor cells. Therefore, the inhibition of PARP1 followed by NK cells transplant suppressed leukemogenesis in PDX models. In addition, PARPi were reported to sensitize AML cell lines and primary cells to human tumor necrosis factor- α-related apoptosis-inducing ligand (TRAIL), a key effector molecule of NK cells, by activating the expression of FAS and TNFRSF10B proteins through enhanced the binding of the transcription factor Sp1 to the promoter) [102]. These data open a new scenario for a rationale usage of PARPi in AML in order to maximize the patients’ benefit, including conditions of functional immune response, like minimal residual disease, or in combination with immunotherapies, as immune checkpoint inhibitors, allogeneic stem cell transplantation, NK cell infusion, recombinant TRAIL, small molecules or monoclonal antibodies functioning as receptor agonists that are currently under investigation.

## Preclinical data of currently available PARP inhibitors in acute leukemias

This section summarizes the main preclinical data available regarding the efficacy of different PARPi in acute leukemias.

### Olaparib

Olaparib (AZD-2281, Ku-0059436) is an orally bioavailable and well-tolerated PARPi. Olaparib binds four members of the PARP super family, PARP1-PARP4 [103] and is the most studied PARP inhibitor in acute leukemia cells. Several selective vulnerabilities to olaparib have been demonstrated in AML and ALL molecular subgroups, that will be better described in “Synthetic lethality and PARP inhibitors: a new therapeutic window for acute leukemia?” section. Based on the evidence that olaparib induces NF-κB-mediated upregulation of FAS [104] and that IKKβ inhibition suppresses HR, PARylation and NHEJ [105] in AML cells, the combined inhibition of the two molecules has been tested. Inactivation of the NF-κB pathway through IKKβ inhibition and olaparib potentiated the efficacy of danunorubin in AML cells (*RUNX1-RUNX1T1* rearranged AML cell line, KASUMI1; erythroleukemia cell line, KG1a) and xenograft models (mice transplanted with KG1a cells), both in monotherapy and when used in combination, by increasing apoptosis and reducing cell proliferation [105]. Moreover, olaparib caused synthetic lethality in combination with decitabine in AML models (KG1a; APL cell line, HL-60; *KMT2A-AFF1* rearranged AML cell line, MV4-11; *FLT3*-ITD AML cell line, PL21) by disrupting BER, which was required for repairing decitabine-induced DNA lesions through recruitment of XRCC1 at DNMT1 foci and repair of trapped DNMT1 [106]. Impairment of HR by WEE1 inhibition was instead responsible for the synergism with olaparib in AML (MV4-11; *KMT2A-MLLT3* rearranged AML cell line, MOLM13; *NPM1/DNMT3A* double mut AML cell line, OCI-AML3) and ALL (*ETV6-RUNX1* rearranged pre B-ALL cell line, REH) cell lines, resulting in DNA damage accumulation and cell death by apoptosis, as also confirmed in an AML murine model [107]. The combination also reduced the proliferative and clonogenic capacity of primary AML cells and induced cell death by apoptosis.

### Rucaparib

Rucaparib (Rubraca/AG-0146991) is an orally bioavailable PARPi, with cancer cytotoxicity levels comparable to olaparib [108]. A recent comprehensive characterization of the target kinase landscape of four FDA-approved PARP drugs found that rucaparib inhibits PARP1, PARP2, PARP3, PARP4, TNSK1 and TNSK2 at nanomolar concentrations [109]. Few preclinical studies have evaluated the efficacy of rucaparib in hematological malignancies. Recently, rucaparib has been tested as single agent or in combination with the antimetabolite 5-fluoro-Uracile (5FU) in vitro (*DNMT3A* mut OCI-AML2 and T-ALL RPMI-8402 cells) and in vivo in AML (mice transplanted with *KMT2A-MLLT3* AML primary cells) and ALL (mice transplanted with pre-T-ALL primary cells) showing elevated efficacy [110]. Upon rucaparib treatment, leukemic cells accumulated low levels of DNA damage and delayed cell cycle progression until S phase arrest. The addition of 5FU in S phase-arrested leukemic cells caused the accumulation of massive DNA damages, probably as a consequence of replicative forks collapse [110].

### Niraparib

Niraparib (MK-4827) is an orally bioavailable PARP1/2 inhibitor, also showing cancer cytotoxicity levels comparable to olaparib [108]. Few studies are available on the efficacy of niraparib in acute leukemias. Niraparib, as well as other PARPs inhibitors, was effective against arsenic trioxide (ATO)-resistant acute promyelocytic leukemia (APL) primary cells alone and in combination with hypomethylating agents (azacitidine and decitabine) or high-dose vitamin C (ascorbate) [111]. The synergism was dependent on the role of PARP1 protein in the processing of DNA demethylation. The triple combination of niraparib, decitabine and histone deacetylase inhibitors (HDACi) also showed a synergistic effect on AML cell lines (*FLT3*-ITD cells MOLM-14) with activation of the ATM pathway, increased production of ROS, decreased mitochondrial membrane potential, and induction of apoptosis. The efficacy of the triple combination was confirmed also on primary leukemic cells (de novo AML, secondary AML, T cell prolymphocytic leukemia and Mixed phenotypes ALL) in which the combination induced DNA damages (H2AX marker) and triggered apoptosis (cleaved PARP1 and Caspase 9) [112]. The mechanism behind the efficacy of the combination consists of trapping of PARP1 and DNMT1 to chromatin, acetylation of DNA repair proteins, and downregulation of the nucleosome-remodeling deacetylase (NuRD) complex that converged toward induction of DSBs, resulting in leukemic cell death.

### Veliparib

Veliparib showed modest cytotoxic activity in ALL and AML cell lines in single agent, obtaining a significant reduction in the cell viability only at concentration 5- to 33-fold higher than plasma concentrations achieved in either animals or humans [113–115]. However, it was shown to potentiate the efficacy of the alkylating agent temozolomide (TMZ) in different ALL and AML cell lines and, in particular, in mismatch repair (MMR)-deficient ALL (T-ALL: MOLT4 and HBS2; REH) cell lines with low O6-methylguanine-DNA methyltransferase (MGMT) activity. Indeed, MMR and MGMT system promote the elimination of the modified nucleotides generated by TMZ. In primary leukemic cells proficient for MMR and with heterogeneous MGMT activity, the authors found that the efficacy of veliparib and TMZ depended to the leukemia subtype. Indeed, veliparib did not significantly potentiate TMZ activity in primary ALL leukemia cells but enhanced the growth-inhibitory effects of TMZ in AML primary leukemias [113].

### Talazoparib

Talazoparib is nowadays the most potent PARP inhibitor due to its ability to induce strong PARP trapping [116]. The number of preclinical and clinical studies evaluating the efficacy of talazoparib against acute leukemia is constantly growing. Kohl and colleagues demonstrated a significant antileukemic activity of talazoparib as monotherapy and in combination with decitabine in primary CD34^+^ AML, MDS and CMML samples [117]. The strong efficacy of the drug combination might be related to the generation of highly cytotoxic PARP1-DNA, DNMT1-DNA and PARP1-DNMT1-DNA complexes. Moreover, hypomethylating agents induced downregulation of RAD51, BRCA1, BRCA2, or HR-related genes, FEN1 or FANCD2 [118], which increased PARPi efficacy in AML, including *FLT3*-ITD and complex karyotype leukemic cells [119]. Strong synergism was also found when combining talazoparib with the novel SAHA-bendamustine hybrid, NL101, in AML, both in vitro (MV4-11; HL-60; *RUNX1-RUNX1T1* rearranged AML cell line, KASUMI1) and in the xenograft model (MV4-11 cells). The authors demonstrated that the combination induced cell apoptosis and cell cycle arrest in G2/M phase and promoted DNA damage [120]. Moreover, HDAC inhibitors were shown to enhance PARP1 binding to DSBs and PARP trapping was further exacerbated by combined treatment with talazoparib, resulting in increased apoptosis [121].

## Synthetic lethality and PARP inhibitors: a new therapeutic window for acute leukemia?

PARPi can act through synthetic lethality, whereby genetic DNA repair defects are enhanced by drug-induced defects in a compensatory pathway [122]. Today, it is well established that PARPi are specifically effective in HR-deficient cells harboring, for example, inactivating mutation of the *BRCA1/2* genes [119, 122, 123]. Indeed, PARP inhibition results in an increase in SSBs, that in turn degenerate into DSBs during DNA replication and become highly cytotoxic in BRCA1/2-deficient cells owing to their reduced capacity for DSB repair. Despite the low frequency of *BRCA1*/*2* mutations in acute leukemia, expression changes have been reported. Scardocci and colleagues showed that the median expression of *BRCA1* mRNA in AML samples is lower compared to normal bone marrow [124]. *BRCA1* downregulation was mediated by promoter hypermethylation and was associated with chromosomal aberrations or therapy-related AML. Of note, PARP1 overexpression and BRCA1 proficiency are predictive of resistance to ex vivo olaparib treatment in AML blasts, whereas formation of γH2AX foci is a marker of sensitivity [125].

During the last decades, it has become clear that oxidative stress and genomic instability, due to mutations in DNA repair genes but also to replication stress, sensitize cancer cells to PARP inhibition [126–129]. In particular, a “BRCAness phenotype,” has been defined for those tumors that share with *BRCA1/2* germline-mutated cases an impaired functionality of the HR pathway, despite carrying functional BRCA1 and BRCA2 [130]. The “BRCAness phenotype” has been also described in acute leukemia. Moreover, heterozygous deletion of *PALB2* is a potential marker of HR defects*,* that may also suggest a potential vulnerability to PARP inhibition [81].

### Selective vulnerabilities of PARP inhibitors in acute myeloid leukemia

This section reports data on sensitivity and resistance biomarkers to PARP inhibitors, which are summarized in Table 4.

**Table 4:** List of identified sensitivity and resistance markers to PARP inhibitors in the preclinical setting

Biomarker	Models	Mechanism	PARP Inhibitor	Effect	References
Fusion genes					
*RUNX1-RUNX1T1*	mouse HSCs and primary AML	Downregulation of Rad51, ATM, BRCA1, and BRCA2	Olaparib, veliparib	Sensitive	[131, 132]
*PML-RARA*	mouse HSCs and primary AML	Downregulation of Rad51, ATM, BRCA1, and BRCA2	Olaparib, veliparib	Sensitive	[131]
*KMT2A-MLLT3*	mouse HSCs	HOXA9 overexpression	Olaparib, veliparib	Sensitive in combination with chemotherapy	[131, 133]
*TCF3-HLF*	ALL	MCPH1 downregulation and consequently HR deficiency	Olaparib	Sensitive	[134]
Activated signaling					
*FLT3*-ITD	murine Lin-cKit + BM cells, primary AML	PARP1 downregulation	Olaparib, talazoparib	Resistant	[135]
*FLT3*-ITD	BaF3, MV4-11, murine Lin-cKit + BM cells, primary AML	FLT3i mediates the downregulation of BRCA1/2, PALB2 and RAD51	Olaparib, talazoparib	Synthetic lethal with FLT3i	[136]
*FLT3*-ITD*;Tet2*^−/−^	murine Lin-cKit + BM cells, primary AML	BRCA1 and LIG4 downregulation; inhibition of TGFβR downregulates ATM, BRCA1, BRCA2, DNA-PKcs and LIG4	Olaparib, talazoparib	Sensitive; synthetic lethal with FLT3i + TGFβRi	[135, 137]
*FLT3*-ITD*;Tet2*^−/−^*; Dnmt3a-/-*	murine Lin-cKit + BM cells, primary AML	BRCA1 and LIG4 downregulation	Olaparib, talazoparib	Sensitive	[135]
*FLT3*-ITD*; Dnmt3a*^−/−^	murine Lin-cKit + BM cells, primary AML	PARP1 downregulation	Olaparib, talazoparib	Resistant	[135]
*RUNX1-RUNX1T1* and *KIT* mut	Kasumi-1, human Lin − CD34 + , primary AML	downregulation of BRCA1 and BRCA2 and the DNA-PK	Olaparib	Synthetic lethal with c-KITi	[138]
*JAK2*^*V617F*^	SET2, HEL, PDX	activation of the ATR-Chk1 pathway	Veliparib	Synthetic lethal with busulfan	[139]
Cohesin complex					
*STAG2,SMC1 and RAD21* mutations	U937, mouse HSCs and PDX	Accumulation of dsDNA breaks; stalled replication forks	Talazoparib	Sensitive	[140]
*TP53*					
*Trp53*/*Bcor* mut	mouse HSCs	Not described	Talazoparib, veliparib	Sensitive	[141]
*IDH1/2*					
*IDH1/2* mutations	Primary AML, HCT116	Downregulation of ATM	Olaparib, talazoparib	Sensitive	[142, 143]
*TET1*					
*TET1* expression	T-ALL	Alteration in the expression of different DNA repair and cell cycle genes	Olaparib	Sensitive	[144]

HSCs: hematopoietic stem cells; AML: acute myeloid leukemia; ALL: acute lymphoblastic leukemia; T-ALL: T cell acute lymphoblastic leukemia; BM: bone marrow; ROS: reactive oxygen species; FLT3i: FLT3 inhibitor; c-KITi: c-KIT inhibitor; mut: mutated; PDX: patient derived xenograft; MPN: myeloproliferative neoplasm; CML: chronic myeloid leukemia

#### Fusion genes

**Fig. 5:**
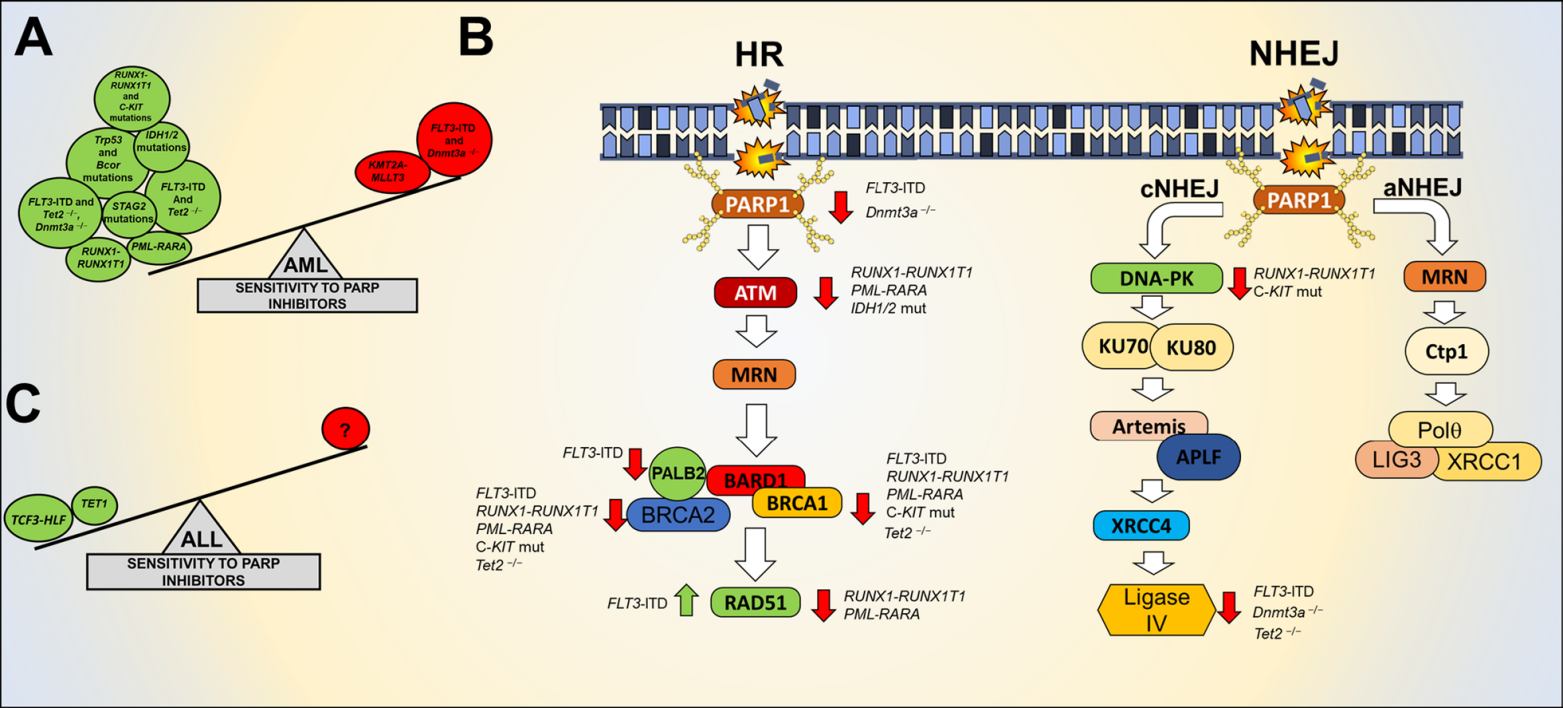
Selective vulnerabilities of PARP inhibitors in AML and ALL. **A** The known molecular alterations that modify the sensitivity of AML cells to PARPi are represented on the two sides of the balance. Green and red indicate molecular alterations enhancing and hampering PARPi sensitivity, respectively. **B** Schematic representation of HR and NHEJ repair pathways in response to DSBs. In the scheme, red and green arrows represent the level of expression of different genes associated with AML subtypes (low and high expression, respectively). **C** The known molecular alterations that modify the sensitivity of ALL cells to PARPi are represented on the two sides of the balance. Green indicates molecular alterations enhancing PARPi sensitivity. To date, no molecular alterations hampering PARP sensitivity have been identified in ALL

Several AML-related molecular alterations, including fusion proteins and mutations, were demonstrated to induce a selective vulnerability to PARPi, through deregulation of the DDR pathway (Fig. 5A and B). Fusion genes are detected in almost 20% of AML patients and usually drive leukemogenesis [145, 146]. Different studies investigated the perturbations deriving from the expression of chimeras in AML and described a relationship between the expression of fusion genes and the response to PARPi. In human CD34^+^ umbilical cord blood cells expressing *RUNX1-RUNX1T1*, genes involved in the FA pathway (*BRCA2*, *FANCA*, *FANCL*, *FANCF*), together with genes in the ATM and ATR and the BER pathways were downregulated, compared with control cells [147]. Consistently, transformed murine hematopoietic cells expressing the *RUNX1-RUNX1T1* chimera displayed a compromised functionality of the HR pathway, as proved by the reduced levels of RAD51, ATM, BRCA1 and BRCA2 [131]. Both murine and human AML expressing the chimera responded to olaparib treatment in vitro and in vivo. Moreover, *RUNX1-RUNX1T1*-driven leukemia is characterized by a mutator phenotype, which implies a predisposition to the acquisition of mutations, both spontaneously and under the pressure of genotoxic agents [148]. This observation has two major consequences: the downside is that chemotherapy can induce the evolution of resistant clones by accumulation of novel mutations, while the upside is that, given their high level of immunogenicity, residual cells may be targeted by combined treatment with PARPi and immunotherapy.

High sensitivity to PARPi was also reported in human and murine models expressing the *PML-RARA* fusion gene [131]. The specificity of the driver fusion protein toward susceptibility to DNA damage accumulation is further supported by the evidence that a *KMT2A* rearrangement induced a modest sensitivity to PARPi. Two independent studies investigated the effect of PARPi inhibition on *KMT2A-MLLT3* rearranged leukemia encoded by the t(9;11)(p22;q23) translocation [131, 133]. Both studies demonstrated a limited sensitivity to PARPi alone. However, Maifrede and colleagues showed that olaparib (in vitro) and talazoparib (in vivo) enhanced the sensitivity of AML cells expressing the *KMT2A-MLLT3* chimera to doxorubicin and cytarabine [133]. From a biological point of view, cell proliferation and differentiation arrest in *KMT2A-MLLT3* leukemia seems to be strictly dependent on resolution of DSBs originated from oxidative stress [149], causing a modest response to PARPi [131]. Indeed, HOXA9-mediated induction of HR genes in *KMT2A-MLLT3*-transformed cells led to the upregulation of DDR target genes such as *RAD51* and *BRCA2* and enhanced resistance to PARP inhibition, that could be reversed by HOXA9 suppression [131]*.*The pressure exerted by antineoplastic agents as combination therapy may cause an accumulation of DSBs until a point of no return [133]. This preclinical evidence suggests that *KMT2A-MLLT3* rearranged patients should not be candidate to PARPi in single agent, as their benefit will likely be modest. In addition, treatment with PARPi was synthetic lethal in proliferating and quiescent AML and ALL cells deficient for DNA-PK–mediated NHEJ [132]. These cases, which can be identified by combined gene expression and mutation analysis, included *BCR-ABL1*-driven leukemias, due to tyrosine kinase-mediated downregulation of BRCA1 and DNA-PKcs protein expression. Taken together, these findings are relevant to the design of personalized therapeutic approaches aided to the eradication of quiescent leukemia stem cells, that are spared by chemotherapy regimens.

#### Activated signaling pathways

Besides chimeric proteins, activating mutations and/or genomic alterations in the signaling pathway, that affect 59% of AML cases [146], were also associated to an augmented HR activity. In *FLT3-*ITD expressing cells, the increased activity of the HR pathway was mediated by the overexpression of RAD51 [150, 151] and the production of ROS [152]. These data were also recapitulated in AML patients carrying *FLT3-*ITD mutation, where the treatment with the FLT3 inhibitor AC220 induced the BRCAness phenotype in *FLT3*-ITD-mutated cells, with downregulation of HR and NHEJ proteins including BRCA1, BRCA2, PALB2, RAD51, and LIG4 and enhanced sensitivity to talazoparib and olaparib [136]. The combination of AC220 and PARPi caused accumulation of lethal DSBs and leukemia cell death. Moreover, it was able to eliminate *FLT3*-ITD quiescent and proliferating leukemia stem cells, as well as leukemic progenitors. A recent study showed that the co-occurrence of *TET2* or *DNMT3A* mutations with *FLT3*-ITD had opposite effect on the response to PARPi. In particular, the co-occurrence of *FLT3*-ITD and *TET2* mutations exacerbated the sensitivity of Lin^−^CD34^+^ primary AML cells to olaparib. On the contrary, cells expressing *FLT3*-ITD alteration alone or in combination with *DNMT3A* mutations were resistant to the treatment with the inhibitor [135]. In line with data form primary samples, mouse models expressing activated-tyrosine kinases (*FLT3*-ITD, *JAK2*^V617F^, *MPL*^W515L^, *NRA*S^G12D^) showed that *Tet2*-deficient cells were sensitive to PARPi, while *Dnmt3a*-deficient cells were not. The combination of talazoparib and quizartinib exerted a strong in vivo inhibitory effect against *FLT3*-ITD^*ITD*^*;Tet2*^−/−^ leukemia, while it was neither effective nor enhanced the effect of the combination in *FLT3*-ITD^*ITD*^*;Dnmt3a*^−/−^ cells. The sensitivity was associated to an increase in lethal DSBs linked to accelerated forks progression and correlated with a reduced expression of BRCA1 and LIG4 proteins in *FLT3*-ITD;*Tet2*^−/−^ and *FLT3*-ITD;*Tet2*^−/−^;*Dnmt3a*^−/−^ compared with *FLT3*-ITD and *FLT3*-ITD*;Dnmt3a*^−/−^ cells. On the contrary, PARP1 was downregulated in *FLT3*-ITD*;Dnmt3a*^−/−^ cells, consistently with reduced HR/cNHEJ and aNHEJ activity, respectively. Finally, regarding *FLT3*-ITD AML, Le et al. described the protecting role of the TGF-β1-TGFβR kinase-SMAD3 pathway in the presence of PARPi in the bone marrow. The use of TGFβR inhibitors in combination with FLT3i in *FLT3*-ITD;*Tet2*^−/−^ mice enhanced the effect of the combination and prolonged the survival of treated mice [137].

The same synthetic lethality effect linked to protein kinase inhibition was also described in *KIT*-mutated AML expressing the *RUNX1-RUNX1T1* fusion gene [138]. The inhibition of c-KIT was associated with downregulation of BRCA1 and BRCA2 and the DNA-PK catalytic subunit (cNHEJ pathway), but not PARP1 (aNHEJ pathway). These led to the restoration of the sensitivity to PARPi. On the same line of reasoning, the combination of veliparib and busulfan was effective in myeloproliferative neoplasm (MPN)/AML xenotransplanted models carrying the activating signaling mutation in *JAK2*^V617F^, where the pharmacological treatment caused G2/M arrest associated with activation of the ATR-CHK1 pathway [139]. Regarding the signaling pathways, the disruption of the tumor suppressor *Pten*, which is targeted by deletions in about 3.0% and 1.0% of pediatric and adult AML, respectively [64], causes centromeric instability and favors spontaneous DSBs [153] which is a marker of PARPi sensitivity.

#### Other genetic subgroups: cohesin or *TP53* or *IDH1/2* mutant AML

A synthetic lethal effect of PARPi was recorded in presence of *STAG2* mutations, a gene encoding for a subunit of the cohesin complex, which regulates sister chromatids during cell division. About 6% of myeloid neoplasm is characterized by the presence of loss-of-function mutations in *STAG2*, representing the most altered gene (51% of cases) among the cohesin family members [154]. In particular, *STAG2* mutations promoted high levels of DNA damage and sensitivity to PARP inhibition [140] in AML. Genome-scale CRISPR/Cas9 screening on the U937 line showed that *STAG2*-mutated cells were preferentially dependent on members of the base excision repair (*PARP1*), homologous recombination (*BRIP1*, *RAD51B*, *RAD51C*, *RAD54L2*, *XRCC2*, *XRCC3*, *PARP1*), mismatch repair machinery (*MSH2*, *POLD3*, *EXO1*) and DNA replication (*RPA2*, *POLD3*), in order to avoid a massive accumulation of genomic instability. The loss of *STAG2* was associated with an increase in stalled replication fork and, therefore, sensitivity to PARPi, that was confirmed in vitro and in vivo*.* Notably, U937 and K562 cells bearing inactivating mutations in *SMC1* or *RAD21*, other two members of the cohesin complex, responded to PARPi to a similar extent of *STAG2*-mutated cells [140]. The synthetic lethal interaction between *STAG2*-mutant cells and DNA damage repair genes was common to other cancer types carrying *STAG2* mutation [155].

Sensitivity to PARPi was also reported in the presence of *TP53* mutations, thus opening a novel potential therapeutic window for a subgroup of high-risk patients [141]. In acute erythroblastic leukemia, data from multiplexed genome editing of mouse hematopoietic stem and progenitor cells and transplant assay showed that *Trp53*/*Bcor*-mutant tumors (carrying wildtype *Dnmt3a* and *Tet2*) were highly sensitive to talazoparib and veliparib, independently of *BRCA1/2* status.

Finally, it has been demonstrated that 2-hydroxyglutarate, the oncometabolite produced by *IDH1/2*-mutated enzymes impaired the HR pathway. Indeed HEL cells expressing mutant *IDH1/2* exhibited levels of DSBs repair defects comparable to those detected in *BRCA1/2*-mutated cell lines and were sensitive to olaparib [143] and talazoparib [142]. Further studies linked the HR defects to the signaling downstream ATM, which was impaired in *IDH1*^R132^-mutated AML [156]. In details, ATM expression was downregulated, due to the elevated methylation of the repressive histone mark H3K9 that may rely on inhibition of the histone demethylases KDM4A and/or KDM4B by 2-hydroxyglutarate [143, 156]. Moreover, daunorubicin enhanced the efficacy of PARPi in *IDH1*/*2*-mutated AML, while IDH1/2 inhibitors antagonized with PARPi and daunorubicin [142].

### Selective vulnerabilities of PARP inhibitors in acute lymphoblastic leukemia

Recent studies revealed that PARP enzymes are highly expressed in T-ALL patients and regulate the expression and post-transcriptional modification of the *TET1* gene [144, 157, 158]. For this reason, *TET1* expressing T-ALL cells are highly sensitive to olaparib (Fig. 5C). From a biological point of view, olaparib abrogated leukemic growth of T-ALL cells in vivo by antagonizing TET1 activity [144]. Selective vulnerabilities driven by fusion genes have been also identified in ALL. In addition to *BCR-ABL1*-mediated suppression of HR and NHEJ [132], Piao and colleagues showed that ALL cells carrying the *TCF3*-*HLF* chimera were hypersensitive to olaparib, both in vitro and in vivo in combination with temozolomide (Fig. 5C)*.* This sensitivity is related to HR deficiency in TCF3-HLF expressing ALL cells. Indeed, the Microcephalin 1 (*MCPH1*) gene, that encodes for a G2/M cell cycle checkpoint regulator, is downregulated by the TCF3-HLF fusion protein resulting in the attenuation of HR activity and in the upregulation of the anti-apoptotic factor BCL2, which suppresses HR activity by interfering with BRCA1 [134].

## Clinical activity of PARP inhibitors in acute leukemia

Overall, in the setting of acute leukemia, only preliminary clinical data on PARPi exist. Ongoing or finished clinical trials are summarized in Table 5.

**Table 5:** Ongoing clinical trials evaluating PARPi in acute leukemia, together with patient population, relevant inclusion criteria that may drive future clinical development, preliminary results

NCT ID	NCT03953898	NCT02878785	NCT03974217	NCT01399840	NCT01139970	NCT00588991	NCT03289910	NCT04207190
Title	Using the Anticancer Drug Olaparib to Treat R/R AML or MDS With an *IDH* Mutation	Decitabine and Talazoparib in Untreated AML and R/R AML (1565GCC)	Talazoparib for Cohesin-Mutated AML and MDS With Excess Blasts	Study of BMN 673, a PARP Inhibitor, in Patients With Advanced Hematological Malignancies [172]	Veliparib and Temozolomide in Treating Patients With Acute Leukemia [160]	Veliparib and Topotecan W/Wo Carboplatin in Treating Patients With R/R Acute Leukemia, High-Risk MDS, or Aggressive MPDs	Topotecan Hydrochloride and Carboplatin W/Wo Veliparib in Treating Advanced MPDs and AML or CML [161]	Talazoparib and GO for the Treatment of CD33^+^ R/R AML
Phase	2	1	2	1	1	1	2	1/2
Number of pts	94	171	12	33	66	12	60	20
Drug and schedule	Olaparib PO BID	Sequential dose escalation of decitabine and talazoparib, 3 + 3 design	Talazoparib, allowed HU	BMN 673	Veliparib PO once daily QD on d 1–4 and twice daily on d 4–12 and temozolomide PO QD on d 3–9 of course 1. Beginning at least 30 d after the start of treatment, pts receive veliparib PO BID on d 1–8 and temozolomide PO QD on d 1–5. Courses. dose escalated	Veliparib when given together with topotecan hydrochloride w/wo carboplatin, doses escalated	ARM A: veliparib orally (PO) twice daily (BID) on days 1–21 and topotecan hydrochloride IV continuously over 24 h and carboplatin IV continuously over 24 h on d 3–7 ARM B: no veliparib	Pts receive talazoparib PO daily on d 1–21 and GO IV over 2 h on d 1, 4, and 7 or d 1 for pts at CR/CRi after cycles 1 or 2. Dose escalated
Population	R/R AML	R/R AML in phase 1, new diagnosis AML unfit phase 2	R/R AML, secondary AML or R/R MDS RAEB after aza	AML, MDS, CLL, MCL	R/R AML, R/R b-ALL, CML in accelerated/blast phase after 2 TKI, CMML-2, new onset high-risk AML > 60 y, new onset high-risk ALL > 60 y	Aggressive MPD or AML out of MPD	New onset AML secondary to MPD, R/R AML secondary to MPD, accelerated phase MPD	R/R AML
Genetics	*IDH1/2 mut*	No	Cohesin mut (*STAG2, SMC1A, RAD21, PDS5B, SMC3*)	No	No	No	No	CD33 > 0.1%
Endpoint	ORR	Safe dose phase 1, CR + CRi phase 2	> 50% blast reduction	MTD	MTD	MTD	ORR	MTD phase 1; ORR extension
Exclusion for cytopenia	No	No	No	BM cellularity < 25% for AML; MCL and CLL: platelet count < 50.000/mm3, neutrophil count < 1.000/mm3	No	No	No	No
Previous sct excluded	No	No	No	No	No	No	No	No
Exclusion for hyperleuko cytosis	> 50.000	> 50.000	> 10.000	> 50.000	> 30.000	> 50.000	No	No
Corrected QT exclusion criteria	> 500	No	No	No	No	No	No	No
Strong CYP3A4 inhibitor	Yes	No	No	No	No	No	No	No
Safety profile for future development	Nr	Nr	Nr	Suitable	Suitable	Nr	Suitable	Nr
Efficacy results	Nr	Nr	Nr	Stable disease in 12/24 pts. AML/MDS, 1 MDS pt. transfusion indpt	CR + CRi 16.6% (8/48); median OS = 5.3 m	Nr	ORR = 33% (14 CR, 11 CRi)	Nr

ALL: acute lymphoblastic leukemia, AML: acute myeloid leukemia, aza: 5-azacytidine, BID: *bis per die*, BM: bone marrow, CLL: chronic lymphocytic leukemia, CML: chronic myeloid leukemia, CMML: chronic myelomonocytic leukemia, CR: complete remission, CRi: complete remission with incomplete hematological recovery, d: days, h: hours, HU: hydroxyurea indpt; independence, IV: intravenously, m: months, MCL: mantle cell lymphoma. MDS: myelodysplastic syndrome, MPD: myeloproliferative disorder, MTD: maximum tolerated dose, mut: mutation, nr: not reported, ORR: overall response rate, PO: *per os*, pts: patients, QD: *quadam per die*, RAEB: refractory anemia with excess blasts, R/R: relapsed/refractory; sct: stem cell transplantation, w/wo: with or without, y: years

Talazoparib was investigated in adult patients with advanced hematological malignancies, including AML, in a single-agent Phase 1 study (NCT01399840). Thirty-three participants were enrolled: Twenty-one of them were affected by AML and four by myelodysplastic syndromes (MDS). For AML, dose-limiting toxicity (DLT) for single-agent talazoparib was demonstrated at a dose of 200 mg/day; no responses were seen in AML, and a hematological improvement was reported in one patient with MDS. Stable disease was reported in 12 of 24 evaluable patients with AML or MDS [159].

Veliparib has been evaluated in two combinatorial regimens in AML. The combination therapy with temozolomide was tested on 48 patients (NCT01139970). DLT level was reached at 200 mg of veliparib, and the phase 2 trial explored the efficacy of the combination between veliparib 150 mg and temozolomide. Complete remission (CR) was documented in 8/48 patients (16.6%). Of note, responders exhibited a veliparib-induced increase in γH2AX in CD34^+^ cells, as marker of DNA damage accumulation [160]. In an umbrella trial on ALL, AML, and chronic myelomonocytic leukemia patients, veliparib was tested in combination with carboplatin and topotecan (NCT00588991). The selected dose of veliparib for the phase 2 part of the trial was 100 mg body mass index. In the entire cohort, 33% of patients achieved at least partial remission, but the overall response rate was higher in the subset of patients with associated or antecedent MPNs or CMML, reaching 64% (14/22) [161]. DNA damage induction in CD34^+^ leukemia cells was again confirmed by the increase in H2AX phosphorylation. Moreover, FANCD2 monoubiquitination was a positive prognostic factor with the combination proposed in the trial.

Of note, in the investigational clinical trials with PARPi, blood count was ever used as an exclusion criterion, for the intrinsic nature of AML and MDS. PARPi have the class effect of lowering platelets production, being PARP an essential enzyme in the physiology of platelet formation [162]. However, low platelet, that was for the aforementioned reason an adverse event of extreme interest, was never reported as a DLT in studies with available results. AML patients usually have a complete deficiency in platelet formation for bone marrow dysfunction, and their platelet level depends on transfusion. It is important to note that PARPi does not affect platelet function, and thus, it does not augment the risks in transfusion-dependent patients.

Seminal results, that will drive future development, are expected from the combination of PARPi with decitabine (NCT02878785), particularly in the sub-cohort of *TET2* and *TET2/DNMT3A*-mutant AML for their high sensitivity to DNA damage [135], and from target-restricted studies in *IDH1*/*2* mutant (NCT03953898) and in cohesin mutant (NCT03974217) AML. The preclinical evidence reported in *IDH1*/*2* [142, 143, 156] or cohesin-mutated AML patients [140, 155], as well as other subgroups, seems to promise success for molecularly directed trial designs for PARPi single agent and combination regimens.

Despite the clinical efficacy of PARPi in oncology, the scientific community is constantly evaluating the risk/benefit ratio of the inhibition DDR pathways for cancer patients. Indeed, the main raised concern is related to the potential genotoxic effect of PARPi in healthy normal cells. The development of secondary myeloid neoplasm (t-MNs) following the use of PARPi in breast, ovarian and pancreatic cancers highlighted the potential toxicity of PARP1 inhibition in healthy hematopoietic precursors [163, 164]. Indeed, the overall incidence of t-MNs shifts from 0.3 to 1% of patients depending on primary tumor subtypes and PARPi used in the study [165]. Nevertheless, the incidence of t-MNs after PARPi is significantly lower than the number cases of t-MNs following the use of conventional chemotherapy (overall incidence 0.8% to 6.3%) [166, 167].

## Conclusions

Olaparib (2014), Rucaparib (2016), Niraparib (2017) and Talazoparib (2018) are currently FDA approved, each with specific indications, for the treatment of advance ovarian, fallopian tube, peritoneal and Her-negative metastatic breast cancer.

The available data on acute leukemia suggest potential windows for successful PARPi treatment in disease subgroups, defined on the basis of molecular markers and/or DDR activity, and/or in combination with genotoxic or targeted agents or immunotherapy, with a particular attention to the disease stage (e.g., minimal residual disease positivity rather than frank leukemia).

However, we have to take into account the evidence that in solid tumors treatment with PARPi is associated with a significantly higher risk of therapy-related MDS and AML [168] (0.73%; 95% confidence interval [CI]: 0·50–1·07) compared with placebo treatment (0.47%; 95% CI: 0·26–0·85; *p* = 0.026), with a median latency between first PARPi exposure and disease development of 17.8 months (8.4–29.2) [169]. Accordingly, treatment of relapsed disease in a high-grade serous ovarian carcinoma patient affected by a synchronous AML with olaparib-based maintenance therapy and the antileukemic agent azacitidine resulted in a dramatic expansion of malignant myeloid cells after two cycles, which was fatal to the patient [170]. Recent data also suggest that mutations of the DDR genes *TP53*, *PPM1D* and *CHEK2*, that are involved in clonal hematopoiesis, occur with increased frequency in cancer patients that were exposed to treatment, and in particular to platinum or topoisomerase II inhibitors or radiation therapy [171], indicating that DDR gene alterations improve the competitive fitness of the cells under these conditions. Taken together, this evidence indicates that a detailed characterization of the genetic background (including mutations, copy number alterations and translocations) and its subclonal architecture, and of the DDR functionality is crucial to personalize therapy and define acute leukemia patients that will likely benefit of PARPi-based therapeutic regimens.

## Data Availability

Data sharing is not applicable to this article as no datasets were generated or analyzed during the current study.

## Abbreviations

ALL: Acute lymphoblastic leukemia
AML: Acute myeloid leukemia
aNHEJ: Alternative non-homologous end joining
APL: Acute promyelocytic leukemia
BER: Base excision repair
CLL: Chronic lymphocytic leukemia
CML: Chronic myeloid leukemia
cNHEJ: Conventional non-homologous end joining
CR: Complete remission
DLT: Dose-limiting toxicity
DDR: DNA damage response
DSB: Double-strand break
FA: Fanconi anemia
HR: Homologous recombination
MRN: Mre11-Rad50-Nbs1
MDS: Myelodysplastic syndromes
MMR: Mismatch repair
MPN: Myeloproliferative neoplasms
NK: Natural killer
PARP: Poly(ADP‐ribose) polymerase
PARPi: PARP inhibitors
ROS: Reactive oxygen species
SSB: Single-strand break
ssDNA: Single-strand DNA
SSR: Single-strand repair
T-MNs: Therapy-related myeloid neoplasm
TRAIL: TNF-related apoptosis-inducing ligand
